# Shallower Root Spatial Distribution Induced by Phosphorus Deficiency Contributes to Topsoil Foraging and Low Phosphorus Adaption in Sugarcane (*Saccharum officinarum* L.)

**DOI:** 10.3389/fpls.2021.797635

**Published:** 2022-02-15

**Authors:** Ke Yi, Xiaofeng Li, Diwen Chen, Shu Yang, Ying Liu, Xinlian Tang, Guizhi Ling, Zunkang Zhao

**Affiliations:** ^1^State Key Laboratory for Conservation and Utilization of Subtropical Agro-Bioresources, Guangxi Key Laboratory for Sugarcane Biology, College of Agriculture, Guangxi University, Nanning, China; ^2^Institute of Nanfan and Seed Industry, Guangdong Academy of Sciences, Guangzhou, China; ^3^College of Land Resources and Environment, Jiangxi Agricultural University/Key Innovation Center for the Integration of Industry and Education on Comprehensive Utilization of Agricultural Wastes and Prevention and Control of Agricultural Non-point Pollution of Jiangxi Province, Nanchang, China

**Keywords:** sugarcane, dynamic observation room, root spatial distribution, phosphorus deficiency, phosphorus efficiency, topsoil foraging, low phosphorus adaption

## Abstract

Low phosphorus (P) availability in acid soils is one of the main limiting factors in sugarcane (*Saccharum officinarum* L.) production. Reconstruction of the root system architecture (RSA) is a vital mechanism for crop low P adaption, while the RSA of sugarcane has not been studied in detail because of its complex root system. In this study, reconstruction of the RSA and its relationship with P acquisition were investigated in a P-efficient sugarcane genotype ROC22 (R22) and two P-inefficient genotypes Yunzhe 03-103 (YZ) and Japan 2 (JP). An efficient dynamic observation room was developed to monitor the spatiotemporal alternation of sugarcane root length density (RLD) and root distribution in soil with heterogeneous P locations. The sugarcane RSA was reconstructed under P deficiency, and R22 had an earlier response than YZ and JP and presented an obvious feature of root shallowness. Compared with the normal P condition, the shallow RLD was increased by 112% in R22 under P deficiency while decreased by 26% in YZ and not modified in JP. Meanwhile, R22 exhibited a shallower root distribution than YZ and JP under P deficiency, supported by 51 and 24% greater shallow RLD, and 96 and 67% greater shallow root weight, respectively. The ratio of shallow RLD to total RLD in R22 was 91% greater than YZ, and the ratio of shallow root weight to total root weight in R22 was greater than that of YZ and JP by 94 and 30%, respectively. As a result, R22 had a higher shoot P accumulation than YZ and JP, which thereby increased the relative leaf sheath inorganic P concentration (RLPC) by 47 and 56%, relative shoot biomass (RSB) by 36 and 33%, and relative cane weight (RCW) by 31 and 36%, compared with YZ and JP under P deficiency, respectively. We verified the reliability and efficiency of a dynamic observation room and demonstrated that a shallower root distribution contributed to improving topsoil foraging, P acquisition, and low P adaption under P deficiency in sugarcane. Therefore, a shallower root distribution merits consideration as an evaluation trait for breeding P efficient sugarcane genotypes and genetic improvement.

## Introduction

The availability of phosphorus (P), one of the three major essential elements, profoundly affects plant metabolism and growth. Soluble inorganic P (Pi) is rapidly immobilized in the soil due to its high reactivity with cations such as aluminum and iron in acidic soils, hampering P absorption by plants (Rubio et al., 2003). The soil in most sugarcane (*Saccharum officinarum* L.) producing areas is acidic, making P deficiency one of the primary limitations of high yield of sugarcane (Roy et al., 2016; Soltangheisi et al., 2021).

Plants have evolved a suite of adaptive mechanisms in response to low P stress, including the enhancement of the expression of Pi transporters, the induction of secretion of root exudates, such as organic acids and acid phosphatase, and the reconstruction of the root system architecture (RSA) including root morphology, growth angle, and spatial distribution (Péret et al., 2014). RSA is a highly plastic trait that varies among and within species (Liu, 2021). Li et al. (2001) reported that P deficiency increased the root dry weight, number, and length of lateral roots in rice, while Vejchasarn et al. (2016) found that the lateral root length of rice was reduced by P deficiency. Similarly, a previous study reported that the shallower root distribution of *Arabidopsis* was caused by a reduction of primary root length and an increase in lateral roots under limited P conditions (Williamson et al., 2001). However, low P stress stimulated the growth of primary roots, inhibited the formation of lateral roots, and increased root depth in maize (Li et al., 2012). In addition, P deficiency weakened root gravitropism and enlarged the growth angle of lateral roots, leading to basal root shallowness in common bean (Liao et al., 2001). The diversity of RSA under low P stress indicates that RSA plasticity depends on external environmental and internal genetic factors.

The selection of appropriate research methods is crucial for the reliable characterization of RSA under different environments and conditions. Traditional techniques, such as excavating, coring, and trenching are widely used to survey root systems in field research (Yuan and Chen, 2013), but those methods are time, labor, cost-intensive, and destructive. Compared to destructive root sampling, the complete root system can readily be obtained with hydroponics, sand culture, and vermiculite culture, and the root images and root morphology can be analyzed using flatbed scanning techniques and applications (ImageJ or WinRHIZO) (Yang et al., 2010). Similarly, scanning techniques have been used to identify seedling root phenotypes in plane culture, including agar plate culture and filter paper culture (Yang et al., 2021). Spatial modeling techniques based on image scanning have enabled two-dimensional (2D) to three-dimensional (3D) RSA quantification. The most commonly used 3D quantified methods involve stratified growth systems (hydroponics or sand culture) and gel growth systems (gellan gum or agar) combined with an advanced high-throughput automatic imaging system that can capture high precision 3D RSA non-invasively and continuously (Clark et al., 2011). However, the homogeneous distribution of nutrients and differences in culture environments are shortcomings of the above culture methods. Emerging technologies such as neutron radiography, nuclear magnetic resonance imaging, and X-ray computed tomography can be applied to non-invasive root system measurement (Mairhofer et al., 2013), but these methods are restricted by their high cost and radiological hazard. In particular, it is worth mentioning that transparent observation port technologies, including rhizotrons, minirhizotrons, rhizotubes, and rhizoboxes, are increasingly applied to survey root growth in greenhouse or field studies. With non-destructive sampling, the growth and development of the root system can be determined continuously in rhizotrons, and the utility of rhizotron technology has been demonstrated in research of wheat (Manschadi et al., 2006), rice (Price et al., 2002), and oilseed rape (Yuan P. et al., 2016).

Sugarcane is an important sugar and biofuel feedstock crop that provides 80% of the world’s sugar and 40% of its ethanol (Zhang et al., 2018; Wu et al., 2020), and mainly grows in tropical and subtropical regions (Huang et al., 2016). The root system of sugarcane is complex in structure and dense in number. Although it is a fibrous root system, sugarcane roots have different characteristics compared to other gramineous crops such as rice and maize and include two root types, sett roots in the early growth period (seedling stage), and shoot roots in the middle and late growth period (Smith et al., 2005). Sugarcane shoot roots consist of “superficial roots”, “buttress roots”, and “rope roots”. However, few studies have systematically evaluated the RSA of sugarcane. In this study, a dynamic observation system, which in combination with rhizotron and scanning techniques, were developed to quantify RSA in sugarcane.

Breeding P-efficient crops with high P acquisition efficiency (PAE) is one economical and practical strategy to deal with the dilemma of P deficiency (Gao et al., 2020). The general definition of high PAE is the ability of crops to acquire more P from soils when given a certain P supply (Lopez-Arredondo et al., 2014; Wang et al., 2019). P acquisition is more important than P utilization to enhance crop P efficiency when P supply is limited (Wang et al., 2010). It is reported that in pigeon pea and rapeseed, the P-efficient genotype can produce more plant biomass and yield at low P supply due to the higher P acquisition capacity (Fujita et al., 2004; Zhang et al., 2009).

The importance of roots to the exploration and acquisition of soil resources and nutrients is self-evident. Previous studies indicated that more complete uptake of water and nutrients was facilitated by root branching in sugarcane (Smith et al., 2005), and nutrient accumulation and utilization efficiency were greatly influenced by sugarcane root morphology (Zeng et al., 2018; Zhou et al., 2021). Nevertheless, few studies have explored the RSA of sugarcane and its role in P acquisition. In our previous study, genotypic differences in P efficiency under high and low P supply were evaluated using 120 different sugarcane genotypes from eight countries in a field trial, and five P-efficient and five P-inefficient genotypes were screened out (Xia et al., 2017). Furthermore, Liu et al. (2021) reported that P-efficiency in sugarcane under low P stress was mainly reflected in enhanced biomass, P uptake and accumulation, P root-to-shoot transfer ratio, and expression of sugarcane P transporters (*SoPht1s*). However, the response of the RSA to low P availability and its effect on P efficiency are still poorly understood in sugarcane. We investigated the RSA using three genotypes associated with distinct root characteristics grown hydroponically and found significant genotypic differences in the root adaptation strategy to P deficiency (unpublished data). In this study, an efficient dynamic observation room suitable for sugarcane root growth was constructed to further determine the changes of sugarcane RSA under heterogeneous P condition. The response of RSA (including root biomass, length density, and spatial distribution) under low P stress and its substantial effect on topsoil foraging, P acquisition, and biomass were investigated using three sugarcane genotypes. The genotype with a shallower root distribution was demonstrated to have greater P efficiency under low P stress in this study. The results will facilitate the subsequent breeding and genetic improvement of P-efficient sugarcane genotypes.

## Materials and Methods

### Plant Materials and Trial Soil

The sugarcane genotypes used in this study were ROC 22 (R22, P-efficient), Yunzhe 03-103 (YZ, P-inefficient), and Japan 2 (JP, P-inefficient). They had distinct root characteristics in hydroponic culture (Supplementary Figure 1). The trial soil used in this study was severely P-deficient soil with available P as low as 1.51 mg kg^–1^. The soil is sandy fluvial soil, collected from Nanyang town, Nanning, China. The basic physical-chemical properties of the trial soil were as follows: pH 4.6, organic carbon 6.9 g kg^–1^, total nitrogen 0.5 g kg^–1^, total P 0.1 g kg^–1^, total potassium 5.2 g kg^–1^, available nitrogen 63.6 mg kg^–1^, exchangeable calcium 819.1 mg kg^–1^, exchangeable magnesium 217.1 mg kg^–1^, and exchangeable aluminum 41.1 mg kg^–1^. Soil pH was determined using a precision pH meter with a soil-to-KCl solution ratio of 1:2.5 (w/v). Organic carbon was measured by the potassium dichromate (K_2_Cr_2_O_7_-H_2_SO_4_) heating method (titrated with FeSO_4_). Total nitrogen was assayed with the semi-micro Kjeldahl method. Total potassium was measured by the flame photometry method after melting with NaOH. Available nitrogen (alkali-hydrolyzable nitrogen) was measured by the alkaline hydrolysis diffusion method (FeSO_4_ reduction and NaOH hydrolysis). Exchangeable calcium and magnesium were extracted by neutral NH_4_OAc and determined by atomic absorption spectrometry. Exchangeable aluminum was assayed with an exchange-neutralization method (extracted with KCl solution and titrated with NaOH). All the measurement methods were referred to Shen et al. (1996) and Landell et al. (2003).

### Root Observation Room

**FIGURE 1:**
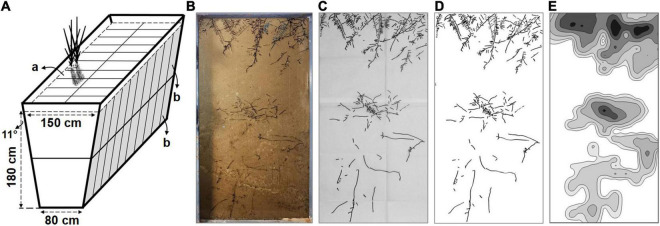
The schematic of the planting container and the processes of root system architecture (RSA) data analysis. **(A)** The structural representation of the planting container and planting troughs. The volume of each planting trough (a) with upper and lower observation glass panel (b) is up to approximately 518 L. **(B)** Outline the root distribution traces with black makers on glass panel. **(C)** Copy the root distribution traces and take pictures of the drawing papers. **(D)** The digitized root image. **(E)** The iso-density curve graph of root length.

The sugarcanes were planted in a root dynamic observation room, which consists of the underground planting container and underground observation aisle. The ground part of the observation room is a solar greenhouse. The external steel framework of the planting container was in the shape of an inverted trapezoid, with an internal depth of 180 cm, an internal bottom width of 80 cm, and an internal top width of 150 cm (Figure 1A). The planting container was evenly divided into separate planting troughs with a plastic septum, and the top of each planting trough is 75 cm long and 50 cm wide. The inner walls on both sides of the planting trough are transparent observation glass panels, which were divided into the upper and lower ports (45 cm width by 85 cm length). The planting troughs were filled with the trial soil and repeatedly irrigated, settled, and filled to ensure the soil bulk density and physicochemical property closer to which at the field site, and then used for experiments as below after 3 months.

### Sugarcane Planting and Experimental Treatment

The seed stems of the sugarcanes were cut into single bud stem segments, and then sterilized and accelerated germination according to the method described by Yang et al. (2019). Eight budding segments were sown evenly in a separate planting trough underground 5 cm close to the glass panel, respectively. The soil moisture content was maintained between 75 and 80% of the field capacity by regularly watering twice a week after sowing, monitored by weighing. The sugarcane seedlings were finally thinned to 4 seedlings with consistent growth when the real leaves could be seen (4 weeks after sowing).

To investigate the low P stress effect on the RSA reconstruction, a lower rate (LP) of 5 mg kg^–1^ P_2_O_5_ was applied to ensure the minimum P demand for sugarcane growth according to our previous field research (Xia et al., 2017). In addition, a normal application rate (NP, 200 mg kg^–1^ P_2_O_5_) was set as a control, and three treatment replicates were carried. Considering the conventional fertilization methods in the field, the P fertilizer was applied evenly into 0–25 cm topsoil at 3 days before sowing seed stem after mixing with nitrogen (50 mg kg^–1^ N) and potassium fertilizers (40 mg kg^–1^ K_2_O). After sugarcane germination, 10 L of 1/5 strength Hoagland’s micronutrient solution was poured into each planting trough. During sugarcane growth, 100 mg kg^–1^ N and 80 mg kg^–1^ K_2_O were also applied to the topsoil at 8 and 16 weeks after sowing, respectively. Sources of P, N, K, and micronutrients were calcium-magnesium phosphate, urea, potassium chloride fertilizers, and chemicals, respectively.

### Samplings and Measurements

To evaluate the effect of low P stress on root distribution and their diversities among genotypes, the root trace images were collected weekly from 10 weeks after sowing, when the root location could be observed in the shallow layer glass panel. Four sampling time points were selected to display the dynamic changes of root distribution (11, 15, 20, and 24 weeks after sowing).

#### Data Collection and Analysis of Root Length Density and Root Length

The root distribution traces on upper and lower observation glass panels were outlined with black markers on the glass panel from week 11 to week 24 (Figure 1B). Then, the traces were copied with black markers on two translucent drawing papers (45 cm by 85 cm) to collect the original root distribution (Figure 1C). Afterward, the drawing papers were photographed at a fixed position with a digital camera (Nikon D700, Tokyo, Japan). Subsequently, the pictures were cropped, stitched, and binarized with ImageJ (Open-source application, National Institutes of Health, Bethesda, MD, United States) to obtain the digitized root images of separate planting troughs (45 cm by 170 cm) (Figure 1D).

The digitized root image was equally divided into 20 sections (each 8.5 cm in height) from top to bottom according to the soil depth. The root length (mm) and the section area (cm^2^) of each section were analyzed with WinRHIZO (Regent Instruments Inc., Quebec, QC, Canada), and the root length density (RLD, mm cm^–2^) of each section was calculated from the root length/section area. Considering that the soil thickness of the cultivated layer in the field is generally 0–20 cm and the depth of sowing in this study is 5 cm, the root systems were divided into shallow and deep layers with a 25 cm soil depth as a boundary with the shallow root ranging from 0 to 25.5 cm (1–3 sections) and the deep root from 25.5 to 170.0 cm (4–20 sections). According to the Pythagorean theorem, the length of a 25.5 cm glass panel is converted to an approximate vertical depth of 25.0 cm at an 11-degree angle of inclination.

Furthermore, the digitized root image was also divided into 600 partitions evenly. Then, each partition was analyzed with WinRHIZO to determine root length. Subsequently, the iso-density curve graph of root length was gridded and constructed with Surfer (Golden Software, Golden, CO, United States) (Figure 1E).

#### Measurements of Plant Height and Weight

The plant length was measured with a wooden ruler from the base of the sugarcane stem to the leaf joint of the last fully expanded leaf (+ 1 leaf) at harvest time, 24 weeks after sowing seed stem. Upper parts of sugarcane were cut off from the base of the sugarcane stem. Canes, stems of the sugarcane, were weighed after removing leaves from stems. The upper parts (shoots) including all of the canes and the removed leaves were collected and weighed after washing with deionized water and drying at 105°C for 1 h and then at 65°C for 3 days. At the same time, roots were extracted from the shallow soil layer (0–25 cm) and deep soil layer (above 25 cm) in each plant trough, respectively. The extracted root samples were weighed after washing and draining. The root: shoot ratio was calculated from the shoot and root biomasses.

#### Phosphorus Measurements

The samples of the leaf sheath were collected from the last fully expanded leaf after weighing the shoots of sugarcane. The Pi in the leaf sheaths were extracted with 200 mM HClO_4_ at 4°C for 10 min and then measured with the molybdenum blue colorimetry method.

The shoot samples were digested with 30% H_2_O_2_ and 98% H_2_SO_4_ after crushing and sieving. P in the digested solution was measured photometrically as described above. The P accumulation was calculated from shoot biomass and P concentration.

#### Soil Phosphorus Content

The soil samples were collected from the 0–25, 25–60, and 60–100 cm soil layers in the planting trough at harvest time. Then, the samples were digested with 72% HClO_4_ and 98% H_2_SO_4_, and extracted with an acidic solution containing 50 mM HCl and 25 mM 1/2 H_2_SO_4_, respectively, for the measurements of soil total P and available P contents. P concentration in the solutions was measured photometrically as described above.

#### Relative Coefficient

Traits related to P efficiency in plants are generally divided into absolute and relative traits, in which the relative traits are commonly used to represent the tolerance of a genotype to low P availability (Xu et al., 2018; Wang et al., 2019; Lin et al., 2020). To present the P nutrition and plant growth, the absolute value and relative coefficient were analyzed in combination in this study. The relative coefficient was defined as % of a certain trait in the LP treatment relative to the NP treatment, consisting of the relative shallow RLD, relative ratio of shallow RLD (Supplementary Figure 3), and P efficiency coefficient (PEC), in which the PEC is also called the relative low P tolerance coefficient, including relative leaf sheath Pi concentration (RLPC), relative shoot P concentration (RSPC), relative shoot P accumulation (RSPA), relative plant height (RPH), relative shoot biomass (RSB), and relative cane weight (RCW).

### Statistical Analysis

The experimental data were analyzed by ANOVA, and the treatment means were compared by Duncan’s multiple range test. Two-way ANOVA was conducted to analyze the effects of P rate, genotype, and their interaction on the RLD, P nutrition, and plant growth. The correlation analysis was expressed with linear regression equation and Pearson correlation coefficient.

## Results

### Phosphorus Content in Different Soil Layers

**FIGURE 2:**
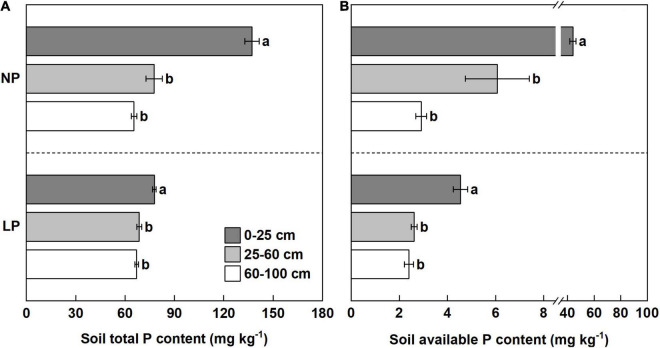
The phosphorous (P) content in different soil layers at harvest time. **(A)** The soil total P content of different soil layers under the lower rate (LP) and normal rate (NP) conditions. **(B)** The soil available P content of the different soil layers under LP and NP conditions. Different lowercase letters indicate a significant difference in the different soil layers within a same P condition (*p* < 0.05), error bars represent standard error of the mean (*n* = 3), according to Duncan’s multiple-range test.

The soil total P content and available P content in the 0–25 cm soil layer were significantly greater than those in the 25–60 and 60–100 cm soil layers, irrespective of the P treatment (LP or NP) (Figures 2A,B). Compared with the 0–25 cm soil layer under the NP condition, the available P content in the 25–60 and 60–100 cm soil layers was reduced by 86 and 93%, respectively (Figure 2B). Furthermore, the available P content in the 25–60 and 60–100 cm soil layers was reduced by 42 and 47%, respectively, compared with the 0–25 cm soil layer under the LP condition (Figure 2B). The results suggest that the location of P is heterogeneous in the planting troughs, and P availability is the greatest in topsoil (0–25 cm).

### Spatial Differences in Root Length Density Under Phosphorus Deficiency Among the Genotypes

**FIGURE 3:**
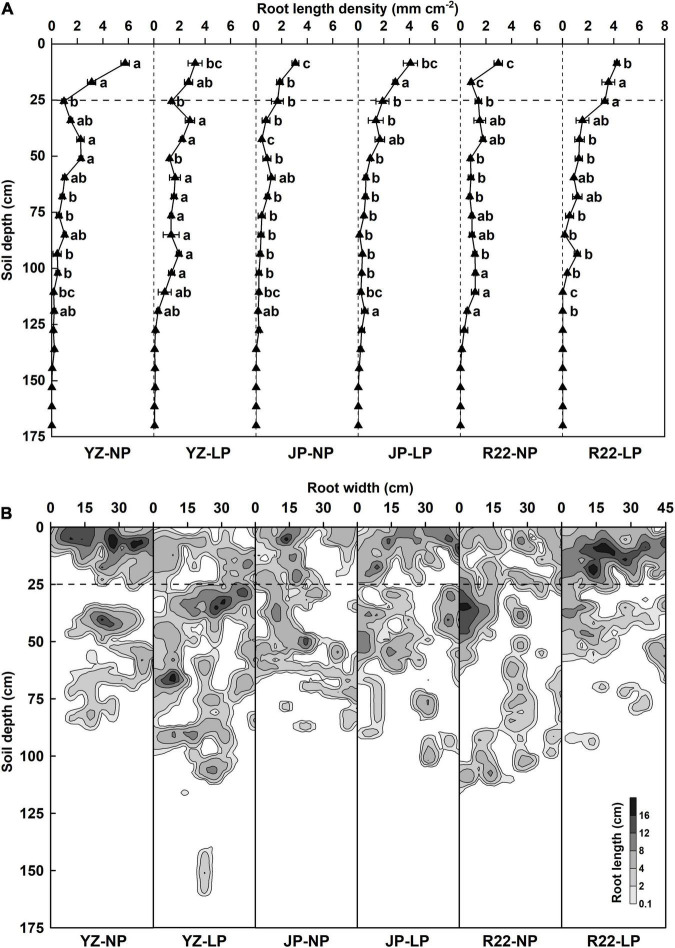
The root length density (RLD) and root distribution under P deficiency at harvest time. **(A)** The RLD of the three sugarcane genotypes YZ, JP, and R22, under the LP and NP conditions. Different lowercase letters indicate significant difference between distinct P conditions and distinct genotypes in the same soil layer (*p* < 0.05), error bars represent standard error of the mean (*n* = 3), according to Duncan’s multiple-range test. The horizontal dotted line in the 25 cm soil depth indicates the boundary of the shallow layer and deep layer. **(B)** The root distribution of the three sugarcane genotypes YZ, JP, and R22, under the LP and NP conditions. The different levels of gray indicate different root lengths.

The RLD and root distribution influenced by P level, genotype, and their interactions were analyzed at harvest time (24 weeks after sowing) (Supplementary Table 1). The total soil depth of 170.0 cm was equally divided into 20 layers (each 8.5 cm in depth) from the top to bottom. In YZ, the LP condition increased the RLD of YZ in the 59.5–76.5 and 85.0–102.0 cm soil layers but reduced the RLD in the 0–8.5 and 42.5–51.0 cm soil layers compared with the NP condition (Figures 3A,B). Conversely, compared with the NP condition, R22 had a greater RLD in the 0–25.5 cm soil layer but a lower RLD the in 93.5–119.0 cm soil layers under the LP condition (Figures 3A,B). Although JP had a greater RLD in the 8.5–17.0 and 34.0–42.5 cm soil layers under the LP condition than under the NP condition, there were no marked differences in the other layers (Figures 3A,B). Meanwhile, under the LP condition, YZ had a greater RLD in the 68.0–102.0 cm soil layers than did JP and R22, respectively (Figure 3A). Similarly, R22 had a greater RLD in the 17.0–25.5 cm soil layers than did JP and R22 under low P stress, respectively (Figure 3A). These results suggest that YZ allocates more roots in the subsoil, while R22 has shallower roots and distributes more roots in the topsoil, and JP exhibited only a mild change in root distribution in response to heterogeneous P-deficient soils.

### Dynamic Changes in Root Length Density Under Phosphorus Deficiency Among the Genotypes

**FIGURE 4:**
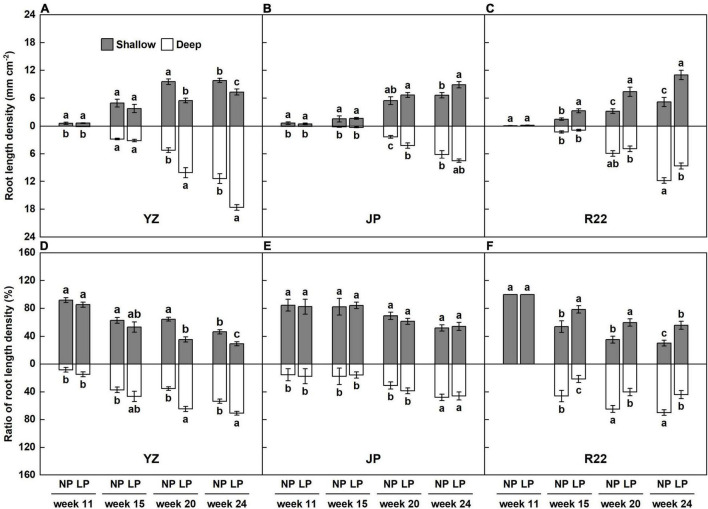
Dynamic changes of RLD under P deficiency. The layered RLD of the three sugarcane genotypes YZ **(A)**, JP **(B)**, and R22 **(C)**. The layered RLD ratio of the three sugarcane genotypes YZ **(D)**, JP **(E)**, and R22 **(F)**. Different lowercase letters indicate significant difference between distinct soil layers and distinct P conditions within a single growth stage (*p* < 0.05), error bars represent standard error of the mean (*n* = 3), according to Duncan’s multiple-range test.

The sugarcane roots were mostly distributed in the shallow layer (83 to 100%) in the seedling stage (week 11), although the shallow root ratio decreased and the deep root ratio increased after entering the tillering stage (week 14). The dynamic changes in root distribution in response to a heterogeneous P-deficient stress differed in the three genotypes (Figures 4D–F and Supplementary Figures 2A–C). In R22, P deficiency induced significant increases of RLD and its ratio in the shallow layer. The percentage increases in the RLD and its ratio reached 120 and 46%, respectively, in week 15. The RLD and its ratio in the shallow layer further increased in R22 after week 20. In week 24, the RLD and its ratio increased by 112 and 86% in R22 (Figures 4C,F). However, P deficiency did not significantly affect the root distribution of YZ up to week 20 (Figures 4A,D). YZ had a 94% greater RLD and 83% greater RLD ratio in the deep layer under the LP condition in week 20. Similarly, P deficiency resulted in an increased RLD and RLD ratio in YZ (by 54 and 32%, respectively) in week 24 (Figures 4A,D). Interestingly, under low P stress, no significant changes in the RLD ratio in JP were found throughout the growth period (Figure 4E), although P deficiency increased the RLD of JP in the deep layer in week 20 and in the RLD in the shallower layer in week 24 (Figure 4B). The above results indicate that root structure can be reconstructed during the early growth period in sugarcane, and this reconstruction in R22 is the rapid response to the heterogeneous P-deficient soils.

### Combined Analysis of Root Length Density and Root Biomass

**FIGURE 5:**
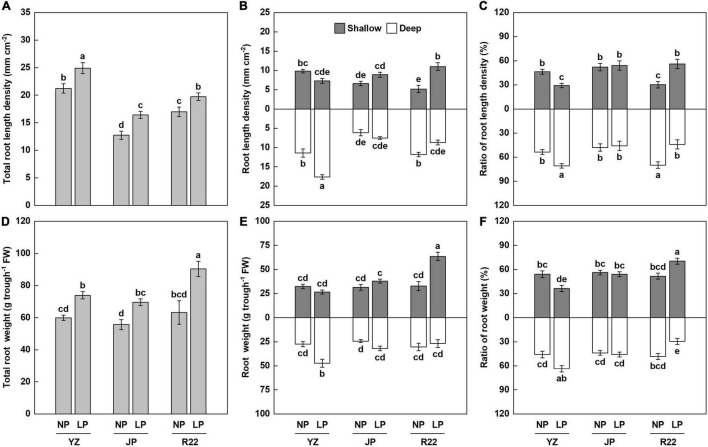
Combined analysis of the RLD and root biomass. The total RLD **(A)**. The RLD **(B)** and RLD ratio **(C)** in the shallow and deep layers. The total root weight **(D)**. The root weight **(E)** and root weight ratio **(F)** in the shallow and deep layers. Different lowercase letters indicate significant difference between distinct P conditions, distinct soil layers and distinct genotypes (*p* < 0.05), error bars represent standard error of the mean (*n* = 3), according to Duncan’s multiple-range test.

In this study, at harvest time (cane elongation stage), the total RLD increased significantly, by 16 to 29%, under the LP condition compared with the NP condition in the three genotypes (Figure 5A). Similarly, P deficiency significantly increased the total root biomass, by 24 to 43%, compared with the NP condition among the three genotypes (Figure 5D). The LP condition also affected root distribution and root weight in various soil layers. Whereas no difference in RLD was found in JP between the LP and NP conditions, the LP condition resulted in an increase in RLD in the deep soil layer in YZ (Figure 5B). However, in R22, the LP condition induced a significant increase in RLD in the shallow layer but decreased the RLD in the deep layer (Figure 5B). Similarly, the LP condition increased the root weight in the deep layer in YZ but increased the root weight in the shallow layer in R22, although there was no difference in root weight in JP between the LP and NP conditions (Figure 5E). Meanwhile, R22 had a 51 and 24% greater RLD and a 96 and 67% greater root weight in the shallow layer compared to YZ and JP under P deficiency, respectively (Figures 5B,E). Also, the LP condition changed the RLD to root weight ratio (Figures 5C,F). In YZ, the LP condition increased the RLD to root weight ratio in the deep layer but reduced it in the shallow layer. Reversely, in R22, the LP condition induced a significant increase in the RLD to root weight ratio in the shallow layer but decreased it in the deep layer. In addition, no difference in the RLD to root weight ratio was found in JP between the LP and NP conditions. Consistent with the results of root dynamic observation, these findings further demonstrate that root shallowing is the response to heterogeneous P-deficient soils in R22 and root deepening is the response in YZ, whereas moderate reconstruction of the root structure occurs in JP in response to heterogeneous soils.

### Tissue Phosphorus Concentration and Phosphorus Accumulation in Plants

**FIGURE 6:**
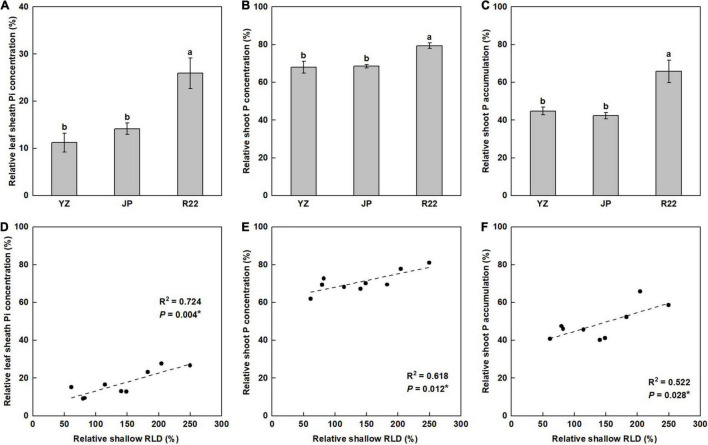
The relative coefficient and correlation analysis of tissue P concentration and accumulation. Relative coefficient of the leaf sheath Pi concentration (LPC) **(A)**, shoot P concentration (SPC) **(B)**, and shoot P accumulation (SPA) **(C)** among the three sugarcane genotypes (the LP condition compared with the NP condition at harvest time). Different lowercase letters indicate significant difference among the three genotypes (*p* < 0.05), error bars represent standard error of the mean (*n* = 3), according to Duncan’s multiple-range test. Correlation of the relative LPC (RLPC) **(D)**, relative SPC (RSPC) **(E)**, and relative SPA (RSPA) **(F)** with the relative shallow RLD. Each point means an individual relative coefficient among the three genotypes and three replicates. Asterisks (*) indicate a significant correlation between two traits at *p* < 0.05, according to the linear regression equation and Pearson correlation coefficient.

Although the leaf sheath Pi concentration (LPC), shoot P concentration (SPC), and shoot P accumulation (SPA) in the three genotypes were reduced by P deficiency, the effects differed among the genotypes and were of lesser magnitude in R22 than in YZ and JP. R22 had a 131 and 83% greater relative LPC (RLPC) than did YZ and JP, respectively (Figure 6A). Similarly, R22 also had a 17 and 16% greater relative SPC (RSPC) and a 47 and 56% greater relative SPA (RSPA) than did YZ and JP, respectively (Figures 6B,C). Furthermore, the RLPC, RSPC, and RSPA were positively correlated with the relative shallow RLD (Figures 6D–F) and its relative ratio (Supplementary Figures 4A–C). Meanwhile, R22 had a 98 and 61% greater LPC and a 38 and 57% greater SPA than did YZ and JP under the LP condition, respectively, although there was no difference in SPC between R22 and YZ (Supplementary Figures 5A–C). The findings demonstrate that a shallower root distribution facilitates P acquisition from topsoil in sugarcane.

### Plant Growth

**FIGURE 7:**
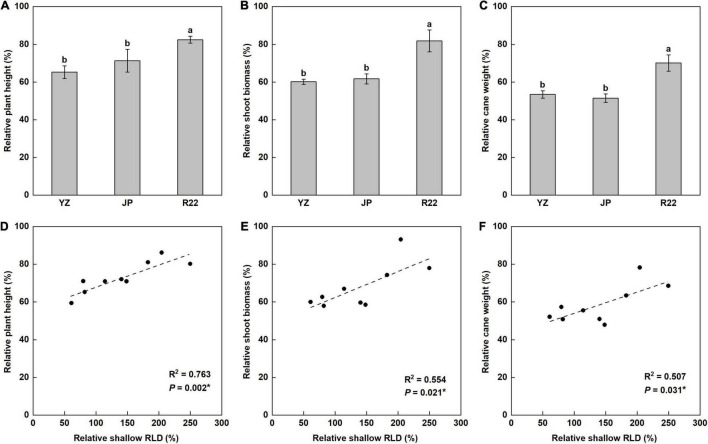
The relative coefficient and correlation analysis of shoot growth. The relative coefficient of the plant height (PH) **(A)**, shoot biomass (SB) **(B),** and cane weight (CW) **(C)** among the three sugarcane genotypes (the LP condition compared with the NP condition at harvest time). Different lowercase letters indicate significant difference among the three genotypes (*p* < 0.05), error bars represent standard error of the mean (*n* = 3), according to Duncan’s multiple-range test. Correlation of the relative PH (RPH) **(D)**, relative SB (RSB) **(E)**, and relative CW (RCW) **(F)** with relative shallow RLD. Each point means an individual relative coefficient among three genotypes and three replicates. Asterisks (*) indicate a significant correlation between two traits at *p* < 0.05, according to the linear regression equation and Pearson correlation coefficient.

Phosphorus deficiency decreased the plant height (PH) by 18 to 35%. However, the decrease in relative PH (RPH) induced in R22 was significantly smaller than those in YZ and JP, and R22 had a 26 and 16% greater RPH than did YZ and JP (Figure 7A). Consistently, the shoot biomass (SB) was decreased by 18 to 40% under P deficiency (Figure 7B). R22 had a 36 and 33% greater relative SB (RSB) than did YZ and JP after LP treatment, but there was no difference in RSB between YZ and JP (Figure 7B). Low P treatment decreased the cane weight (CW) by 47, 49, and 30% in YZ, JP, and R22, respectively. R22 had a 31 and 36% greater relative CW (RCW) than did YZ and JP (Figure 7C). Moreover, the RPH, RSB, and RCW were positively correlated with the relative shallow RLD (Figures 7D–F) and its relative ratio (Supplementary Figures 4D–F). Meanwhile, R22 had a 36 and 23% greater SB and a 58 and 35% greater CW than did YZ and JP under the LP condition, respectively (Supplementary Figures 5E,F). Compared to the NP condition, P deficiency significantly increased the root: shoot ratio, but there was no difference among the three genotypes (Supplementary Figure 6). Additionally, R22 had a 23% greater PH than did YZ under low P stress (Supplementary Figure 5D). These findings suggest that a shallower root distribution enhances plant growth under P deficiency, thereby increasing plant height and cane weight.

## Discussion

Our results demonstrated that a sugarcane shallower root distribution induced by low P stress promoted topsoil foraging, enhanced P acquisition, and improved low P adaption under P deficiency. Under low P stress, R22 (root-shallowing genotype) had a greater RLD and shallower root distribution in topsoil compared with YZ (root-deepening genotype) and JP (root-maintaining genotype) (Figures 3–5). In R22, this promoted growth and adaptability to P-deficient soil compared to YZ and JP, as evidenced by a greater RLPC, RSPC, and RSPA (Figure 6), and therefore, a greater RPH, RSB, and RCW (Figure 7).

### The Efficient Dynamic Observation Room for Examining the Sugarcane Root System

As so-called the “hidden half”, plant roots are typically inaccessible and difficult to observe and measure. Excavating and coring are the traditional techniques used in field research but are time and labor-intensive (Wu and Guo, 2014), thus those techniques are not practical in continuous research. Hydroponics, sand culture, and soil culture are good choices used in greenhouse research because root morphological data can be scanned and analyzed using the image-analysis software, such as ImageJ and WinRHIZO (Kenobi et al., 2017), while the homogeneous distribution of nutrients and differences in the culture environment are also important limitations. Previous studies reported that the sugarcane roots could grow outward and downward into the subsoil to a depth of up to approximately 200 to 600 cm in fields (Smith et al., 2005). Thus, we established a dynamic observation room suitable for sugarcane root growth based on rhizotrons and scanning techniques. The dynamic observation room consisted of a planting container and an underground observation aisle. The planting container was evenly divided into separate planting troughs (Figure 1A). Unlike the less thin soil thickness (3–10 cm) used in a previous study (Manschadi et al., 2006), each planting trough in this study could support a horizontal range of 75 cm, a vertical range of 180 cm, and a total volume of approximately 518 L for the growth of sugarcane roots. The system may also be used for research on other tall crops such as sorghum (*Sorghum bicolor*), maize (*Zea mays*), and cassava (*Manihot esculenta* Crantz). Importantly, the environment of the dynamic observation room is close to the field conditions, which can provide support for the subsequent field research.

In contrast to being positioned at a 45-degree angle (Kuchenbuch and Ingram, 2002), the observation glass panel in this study was formed an angle of 11 degrees with the vertical direction, ensuring that the roots grew along the glass panel, and it had little influence on the actual depth of the root system (Figure 1A). Yuan P. et al. (2016) set an analogous angle (15 degrees) and commendably unraveled the dynamic changes of RSA in oilseed rape. The length deviation decreased according to the angle of the observation glass panel from 41 (45 degrees) to 4% (15 degrees) and 2% (11 degrees) at the same vertical soil depth.

The root distribution pattern was captured *in situ* and non-invasively by copying root traces. The digitized root images were analyzed after collecting data on dynamic changes of the roots (Figures 1B–D). The growth period of sugarcane is 24 weeks, including the germination stage (week 1–4), seedling stage (week 4–14), tillering stage (week 14–20), and cane elongation stage (week 20–24), and the root distribution was recorded weekly from 10 weeks after sowing (Figure 4). We innovatively used the iso-density curve graph of root length (Figure 3B and Supplementary Figures 2A–C) and revealed the spatiotemporal changes in sugarcane root distribution under low P stress. Our results showed that low P stress significantly increased the RLD with the three sugarcane genotypes (Figure 5A). Under the LP condition, R22 had a shallower root distribution, YZ had a deeper root distribution, and no significant change was observed for the root distribution of JP (Figures 5B,C).

The root system data can be described more accurately by combining several methods. Besides the glass panel observation, we further excavated sugarcane roots in the topsoil and subsoil at the cane elongation stage. R22 had a larger root weight and root weight ratio in topsoil than did YZ and JP under P deficiency (Figures 5E,F). The above results of root excavation were consistent with the aforementioned observations (Figures 5B,C), verifying the reliability and efficiency of the dynamic observation room.

### Low Phosphorus Availability Regulates the Root System Architecture in Sugarcane

The RSA is a structural and highly plastic trait that varies among and within species and is regulated by external environmental and internal genetic factors (Péret et al., 2014). It includes the root morphology, root growth angle, and root spatial distribution. One important regulating factor is the availability of P, and the reconstruction of RSA is a vital adaptive mechanism to low P stress (Del Bianco and Kepinski, 2018). Dicot plants such as *Arabidopsis*, soybean, and oilseed rape have a tap root system, while monocot plants have a fibrous root system, such as rice, maize, wheat, and sorghum (Meng et al., 2019). The different RSAs of the two plant groups developed distinct mechanisms for capturing P under low P conditions. Additionally, the RSA can be regulated differently under low P stress in the same species, depending on genetic characteristics, culture conditions, and other plant regulators (Liu, 2021).

Monocot plants, such as rice, facilitate root adaptation to low P condition by increasing root hair elongation, lateral root density, and lateral root length (Li et al., 2001; Giri et al., 2018), while Vejchasarn et al. (2016) found that P deficiency reduced the lateral root density and increased the length and density of root hairs in rice. In wheat, the root hair density and root hair length in low P soil were greater than that in high P soil (Yuan H.M. et al., 2016), similarly P deficiency promoted fine root proliferation to maintaining the stability of total root length (Shen et al., 2018). In this study, low P availability significantly increased the total RLD, total root weight, and root: shoot ratio with three sugarcane genotypes (Figures 5A,D and Supplementary Figure 6), and the lateral root length accounted for more than 95% of the total root length (data not shown). Similarly, in our previous study, to dynamically observe the characteristics of the sugarcane root system, a hydroponics culture device with a special stratified mesh was designed to fix the root system. The results of the hydroponics experiment showed that the root length and root biomass increased significantly under P deficiency, which was mainly due to an increase in lateral root number and density (Supplementary Figure 1). It suggested that root proliferation caused by an increase in the lateral root number is an important adaptive strategy to P deficiency in sugarcane.

The root spatial distribution also responds plastically to low P availability. P deficiency inhibited primary root growth in *Arabidopsis*, enhanced lateral root formation, and led to a shallower root distribution (Williamson et al., 2001), while it was reported that P starvation reduced the lateral root numbers, promoted primary root formation, and increased the whole root depth in maize (Li et al., 2012). In addition, the root gravitropic setpoint angle (GSA) is closely related to P availability. In common bean, the shallowness of basal roots was caused by decreased root gravitropism and an increased GSA of lateral roots under low P condition (Liao et al., 2001). Huang et al. (2018) reported that a rice actin-binding protein (RMD) dampened root gravitropism, increased the crown root angle, and formed shallower roots in response to low P availability. Our results showed that YZ had a greater RLD and its ratio (Figures 4A,D) in the deep layer, whereas R22 had a greater RLD and its ratio in the shallow layer under the LP condition compared to the NP condition at harvest time (Figures 4C,F). Interestingly, although the LP condition increased the RLD of JP in the shallow layer compared to the NP condition (Figure 4B), the RLD ratio of JP in the shallow and deep layers did not increase noticeably under P deficiency throughout the growth period (Figure 4E). Therefore, R22, YZ, and JP had distinct regulatory mechanisms underlying the root response to low P availability, possibly reflecting differences in root function and P efficiency. Thus, we defined R22 as the root-shallowing genotype, YZ as the root-deepening genotype, and JP as the root-maintaining genotype according to the different responses of their RSA to low P availability.

Improving crop PAE is an effective strategy to deal with P deficiency. As the principal organ by which plants absorb nutrients, the roots and RSA are closely related to PAE (Péret et al., 2014; Liu, 2021). Our results showed that R22 had a greater RLD, shallower root distribution, greater root weight, and higher root biomass ratio in topsoil than did YZ and JP under low P stress (Figure 5). R22 also had the highest relative shallow RLD and its relative ratio among the three genotypes (Supplementary Figures 3A,B). Moreover, the response of R22 to P deficiency was more rapid compared to YZ and JP (5 weeks earlier) (Figure 4C), indicating that R22 exhibits better adaptation to low P availability. In our previous hydroponics experiment (Supplementary Figure 1), R22 had a shallower root distribution, greater root width, and larger root weight ratio in the shallow layer than YZ and JP under the LP condition. Therefore, we obtained comparable results from the hydroponics experiment (homogeneous P conditions) and the dynamic observation room (heterogeneous P conditions). Low P promoted sugarcane root proliferation and R22 had the highest shallow root distribution ratio, indicating that root shallowness is a significant feature of R22 under low P stress.

### Shallower Root Spatial Distribution Contributed to Phosphorus Acquisition and Adaptation to Low Phosphorus

Soil available P is the most effective part of soil P storage for crops, and it is an important index with which to evaluate the level of soil P supply (Ticconi and Abel, 2004). The migration ability of P in the soil is weak, and the applied P fertilizer is rapidly fixed by the soil to form insoluble compounds (Rubio et al., 2003). Thus, P availability is the greatest in the topsoil and lower in the subsoil, and shallow root growth angles favor topsoil foraging and P capture (Lynch, 2011). In this study, our results showed that the distribution of P in trial soil was heterogeneous, and that the soil total P content and soil P availability in the topsoil (0–25 cm) were significantly higher than those in the subsoil (25–100 cm) (Figure 2). Therefore, the more roots in shallow soil, the more likely roots will be exposed to P.

The advantage of a shallow root architecture for topsoil foraging has been demonstrated previously in soybean, common bean (Lynch and Brown, 2001), and maize (Zhu et al., 2005). Topsoil foraging is an efficient adaptation of plant roots to low P availability based on enhanced root exploration, P acquisition, and yield production, and the primary changes include a larger vertical root angle and shallower root system (Lynch and Brown, 2001). In this study, a shallower root spatial distribution contributed to improving topsoil foraging, P acquisition, and low P adaptation. Under the LP condition, R22 exhibited improved LPC, SPA, RLPC, RSPC, and RSPA compared to YZ and JP (Figures 6A–C and Supplementary Figures 5A,C). The positive correlation between the relative shallow RLD and its relative ratio with the RLPC, RSPC, and RSPA indicated that a greater shallow RLD and shallower root distribution increased the exploration of roots to P and promoted more root exploitation in the topsoil, resulting in greater P acquisition from the topsoil (Figures 6D–F and Supplementary Figures 4A–C). Similarly, Liao et al. (2001) found that P acquisition increased and the adaptation to low P availability was correlated with shallower roots and a greater root growth angle with a P-efficient genotype of common bean. Zhao et al. (2004) reported that a shallow root architecture led to greater P efficiency and soybean yield. Furthermore, in this study, R22 exhibited improved adaptation to P deficiency, as evidenced by the greater SB, CW, RPH, RSB, and RCW compared with YZ and JP (Figures 7A–C and Supplementary Figures 5E,F). The RPH, RSB, and RCW were positively correlated with the relative shallow RLD (Figures 7D–F) and its relative ratio (Supplementary Figures 4D–F), indicating that low P availability had the least effect on R22, and a greater shallow RLD and shallower root distribution promoted adaption to low P condition. In general, our results indicate that a shallower root distribution would enhance the adaptation of sugarcane to low P stress, increase P acquisition from the topsoil, and improve growth under P deficiency. Therefore, we suggest that the presence of a shallower root distribution merits consideration as an evaluation indicator for breeding P-efficient sugarcane genotypes and genetic improvement.

The RLD is strongly associated with crop nutrient and water uptake (Ning et al., 2019), whereas root biomass reflects plant carbon allocation and soil carbon dynamics (Hirte et al., 2018). In this study, the RLD under the LP condition at harvest time was in the order YZ > R22 > JP (Figure 5A), whereas root biomass was in the order R22 > YZ = JP (Figure 5D), indicating that YZ and R22 adopted different adaptation strategies to balance carbohydrate allocation and nutrient absorption under low P stress. Previous studies in maize have shown that reduced lateral root branching also reduced the metabolic cost of soil exploration, promoting axial root elongation and increasing rooting depth, which improved water and nitrogen (N) capture under water and N stress conditions (Zhan et al., 2015; Zhan and Lynch, 2015). Our results showed that root shallowness is momentous for P acquisition and plant growth (Figures 6, 7). R22 had a greater RLD and RLD ratio in the shallow layer, which led to a shallower root distribution and improved exploration of topsoil (Figures 5B,C). R22 had an equal root: shoot ratio with JP and YZ under P deficiency, suggesting that more root proliferation also promoted the increase of shoot biomass in R22 (Supplementary Figure 6). Although larger root biomass is accompanied by greater carbohydrate metabolism, the overall benefits of P absorption and cane production brought by root shallowness are greater than the energy consumption for root proliferation.

Commercial sugarcane varieties were mostly hybridized from crosses between *S. officinarum* and *S. spontaneum* (Thirugnanasambandam et al., 2018). The root features of sugarcane depend on the proportion of genomic introgression of *S. spontaneum* (Jackson, 1994). There are three types of sugarcane shoot roots: “superficial roots”, “buttress roots”, and “rope roots” (Smith et al., 2005). Superficial roots are typically located horizontally in the topsoil and are the principal absorbing roots. Previous studies reported that 65% of the root biomass at harvest was concentrated in the topsoil (20 cm depth) (Otto et al., 2009). In this study, the RLD ratio in the top 25 cm soil layer ranged from 30 to 56% (Figure 5C), and the root weight ratio ranged from 39 to 70% (Figure 5F). Under the LP condition, R22 had a greater RLD ratio and root weight ratio compared to YZ and JP, which promoted P acquisition and increased cane weight (Figures 6, 7). Besides, in our previous study, we found that R22 had a shallower root distribution and lower root depth by the hydroponics culture device (Supplementary Figure 1). These results implied that the root characteristics of “superficial roots” of R22 conferred the greater P acquisition and better growth to a great extent, compared with YZ and JP.

In addition, rope roots are a typical characteristic of *S. spontaneum* and can penetrate the soil profile vertically to a depth of 600 cm (Smith et al., 2005). In our previous study using the hydroponics culture device, greater root depth and deeper root distribution were found in YZ compared with R22 (Supplementary Figure 1). The roots of YZ are vertically distributed with a narrower root width, similar to the rope roots of *S. spontaneum* (unpublished data). In this study, the RLD and root weight of YZ in the deep layer increased under the LP condition (Figures 5B,E), increasing the RLD ratio and deepening the root distribution in subsoil (Figures 5C,F). Water and nitrate (N) are usually concentrated in the deeper subsoil due to their high mobility. An increased RLD in the subsoil improves water and N acquisition and plant growth (Zhan and Lynch, 2015; Gao and Lynch, 2016). Therefore, YZ, which harbors the root-deepening genotype, possibly has notable water and N acquisition capabilities. In short, the genetic overlap of R22 and YZ with *S. spontaneum* and the substantial influence of the genetic constitution warrant further study.

Although the complex soil environment and genetic background have hampered research on the sugarcane root system, the breeding of appropriate root phenotypes can optimize sugarcane production. Furthermore, metrics associated with the RSA are feasible evaluation indicators for breeding efficient genotypes and the genetic improvement of sugarcane.

## Conclusion

A dynamic observation room for sugarcane root growth was established and its reliability and efficiency were verified. Root proliferation is an important adaptive strategy to P deficiency in sugarcane, and the regulatory mechanisms of the RSA response to low P availability differed among three genotypes. Furthermore, we defined the sugarcane genotype with a greater shallow RLD and shallower root distribution under a low P condition as the root-shallowing genotype. Moreover, our results dissect that the shallower root distribution induced by low P stress promoted topsoil foraging, enhanced P acquisition, and improved low P adaption under P deficiency in sugarcane, as evidenced by greater P acquisition from the topsoil and enhanced plant growth and cane production. Therefore, a shallower root distribution merits consideration as an evaluation trait in the breeding of P-efficient sugarcane genotypes and for genetic improvement.

## Data Availability Statement

The original contributions presented in the study are included in the article/Supplementary Material, further inquiries can be directed to the corresponding author.

## Author Contributions

KY performed the research, conducted the experiments, and wrote the manuscript. ZZ and XL designed the research, supervised the study, and revised the manuscript. DC and SY analyzed the data. YL, XT, and GL participated in the study’s design. All authors read and approved the final manuscript.

## Conflict of Interest

The authors declare that the research was conducted in the absence of any commercial or financial relationships that could be construed as a potential conflict of interest.

## Publisher’s Note

All claims expressed in this article are solely those of the authors and do not necessarily represent those of their affiliated organizations, or those of the publisher, the editors and the reviewers. Any product that may be evaluated in this article, or claim that may be made by its manufacturer, is not guaranteed or endorsed by the publisher.
