# Supplementary material for: Shallower Root Spatial Distribution Induced by Phosphorus Deficiency Contributes to Topsoil Foraging and Low Phosphorus Adaption in Sugarcane (Saccharum officinarum L.)
Source: Front Plant Sci. 2022 Feb 15;12:797635. doi: 10.3389/fpls.2021.797635 (PMC8887604; doi:10.3389/fpls.2021.797635)
Supplement: Supplementary file 1 [file Data_Sheet_1.docx]

Supplementary Material

**Supplementary Table 1.** Summary of ANOVAs for RLD in different soil layers as influenced by P level (P), Genotype (G), and their interactions (P×G).

| **Soil layer** | **P** | **G** | **P×G** | **Soil layer** | **P** | **G** | **P×G** |
| --- | --- | --- | --- | --- | --- | --- | --- |
| **1** | 0.06 | 0.49 | 0.72* | **11** | 9.66* | 11.67* | 9.35* |
| **2** | 0.82* | 0.98 | 0.66* | **12** | 0.16 | 10.83* | 15.4* |
| **3** | 0.57* | 0.99* | 0.09* | **13** | 0.55 | 1.24 | 7.14* |
| **4** | 0.77 | 0.27 | 0.19 | **14** | 0.02 | 0.15 | 5.63* |
| **5** | 0.41 | 0.90* | 0.53* | **15** | 0.06 | 1.84 | 0.42 |
| **6** | 0.51 | 0.32* | 0.50* | **16** | 0.19 | 0.51 | 2.80 |
| **7** | 0.03 | 0.38 | 0.22 | **17** | 1.32 | 0.90 | 0.35 |
| **8** | 0.19 | 0.97 | 0.92* | **18** | 1.00 | 1.00 | 1.00 |
| **9** | 0.80 | 0.35 | 0.18* | **19** | 6.04 | 2.82 | 2.82 |
| **10** | 0.92 | 0.56* | 0.78 | **20** | 1.41 | 0.85 | 0.85 |

The associated F-values and probabilities (*P < 0.05) were shown.


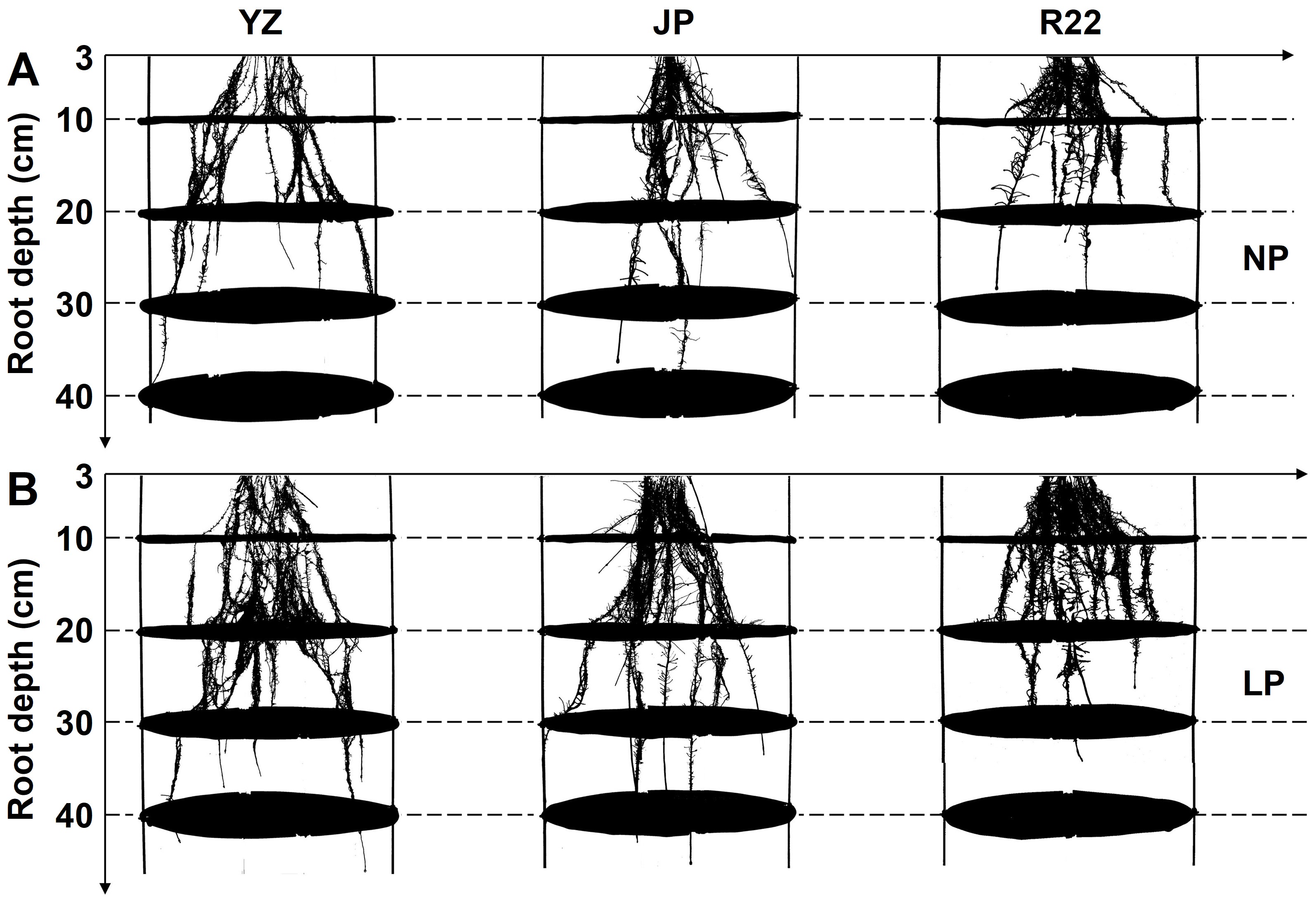


**Supplementary Figure 1****.** The root adaptation strategy of the three sugarcane genotypes. Root morphology and distribution under normal P (NP) **(A)** and low P (LP) **(B)** conditions in the hydroponics experiment.


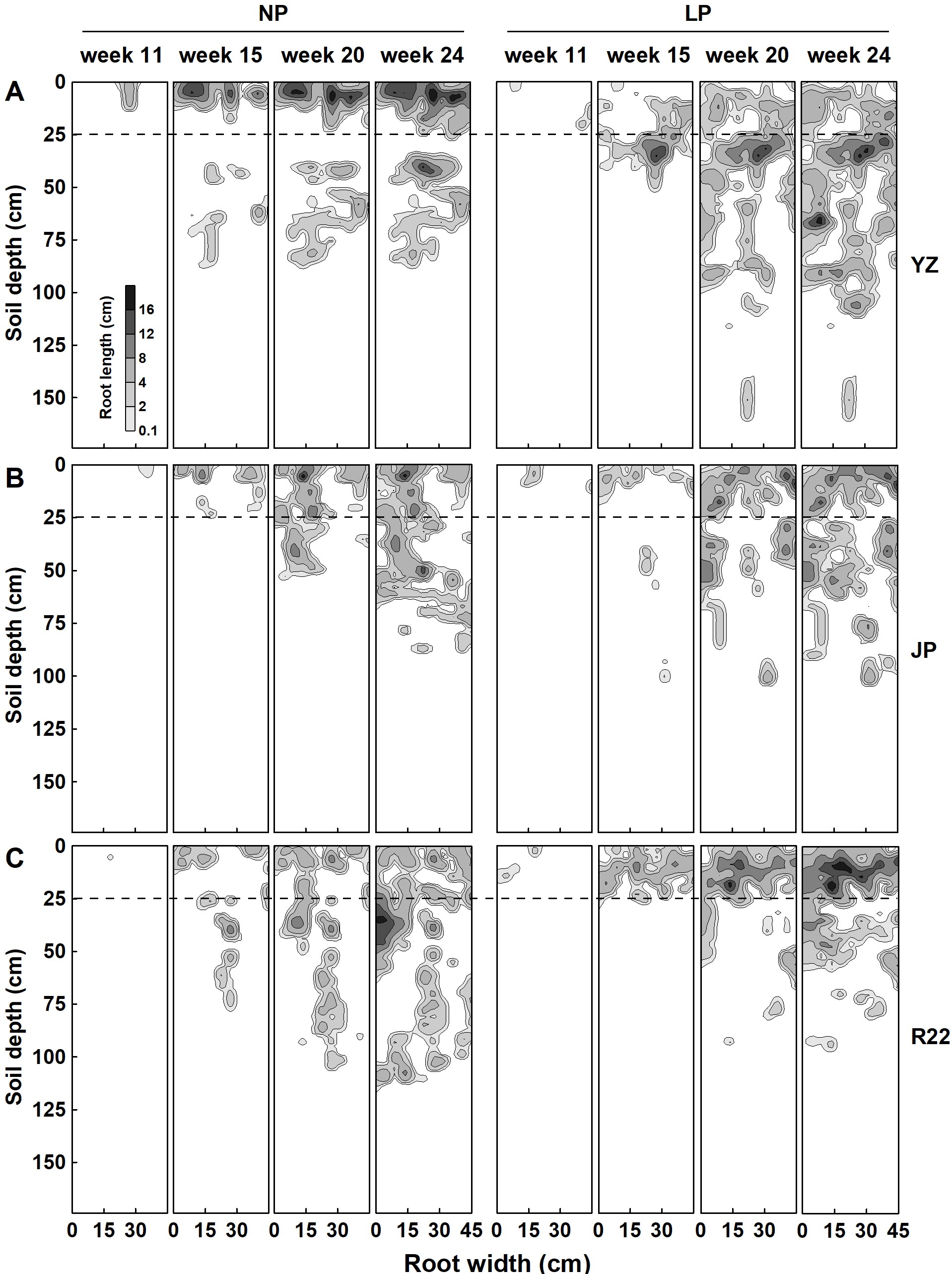


**Supplementary Figure 2.** The iso-density curve graph of root length in different growth stages. Continuous change of the root spatial distribution of three genotypes YZ **(A)**, JP **(B)** and R22 **(C)**, under the LP and NP conditions in different growth stage. The dotted line in the 25 cm soil depth indicates the boundary of the shallow layer and deep layer. The different levels of gray indicate different root lengths.


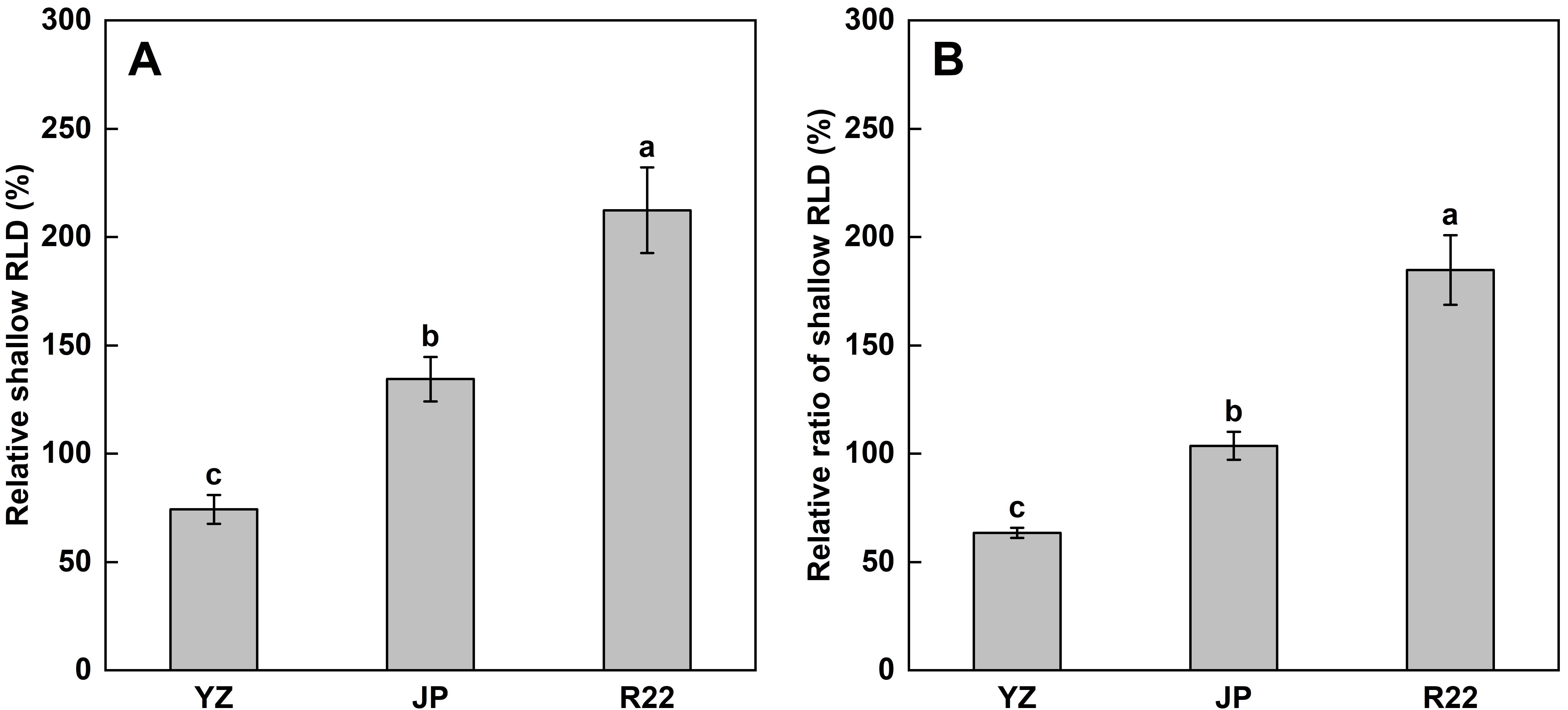


**Supplementary Figure 3.** The relative coefficient of the shallow RLD and shallow RLD ratio. The relative shallow RLD **(A)** and relative shallow RLD ratio **(B)** among the three sugarcane genotypes (LP condition compared with NP condition). Different lowercase letters indicate significant difference among the three genotypes (P < 0.05), error bars represent standard error of the mean (n = 3), according to Duncan’s multiple-range test.


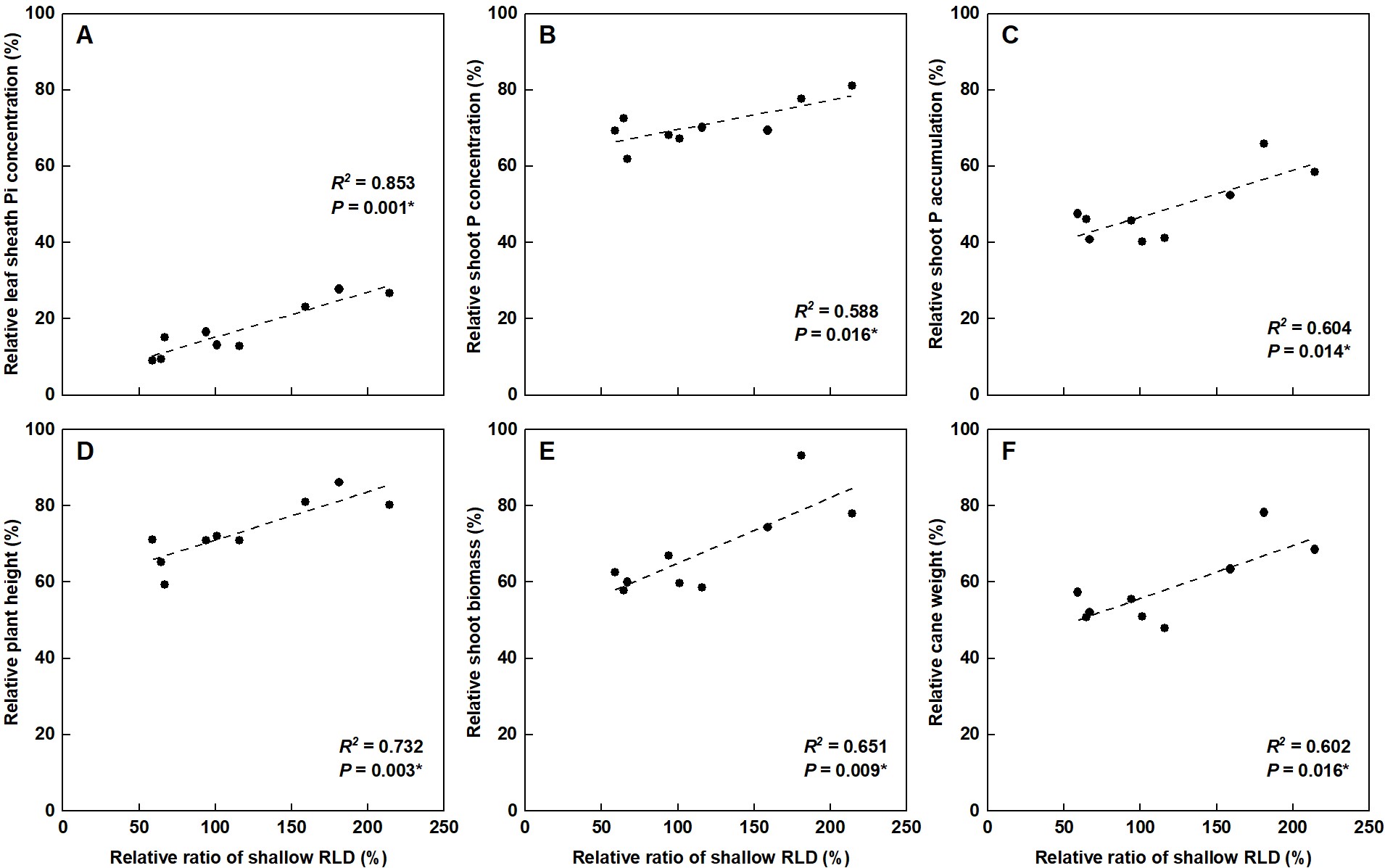


**Supplementary Figure 4.** Correlation analysis of the relative coefficients. Correlation of the RLPC **(A)**, RSPC **(B)**, RSPA **(C)**, RPH **(D)**, RSB **(E)** and RCW **(F)** with the relative ratio of shallow RLD. Each point means an individual relative coefficient among the three genotypes and three replicates. Asterisks (*) indicate significant correlation between two traits at P< 0.05, according to the linear regression equation and Pearson correlation coefficient.


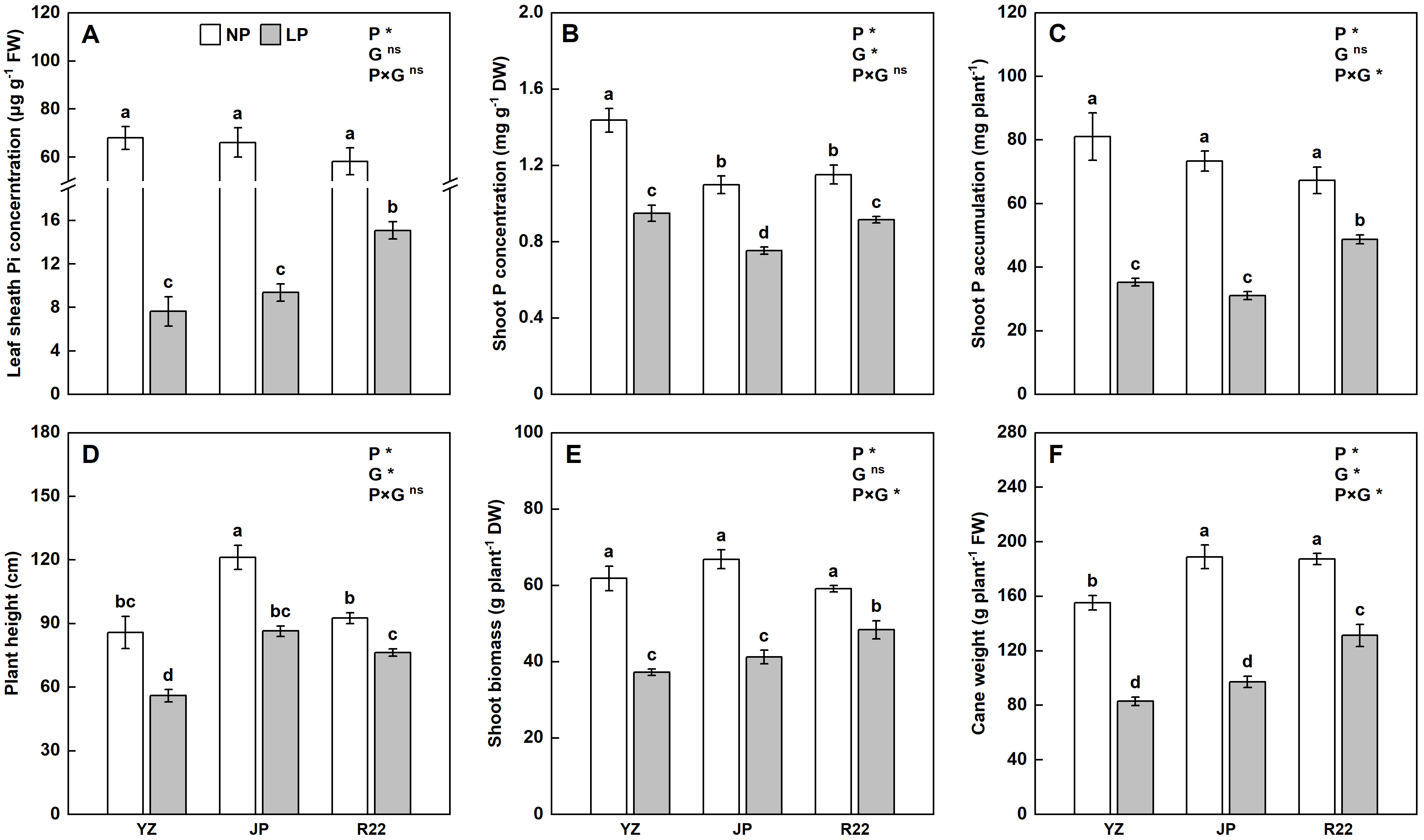


**Supplementary Figure 5.** The tissue P concentration, P accumulation, and plant growth. The LPC (**A**), SPC (**B**), SPA (**C**), PH (**D**), SB (**E**), and CW (**F**) among the three sugarcane genotypes under the LP and NP condition. Different lowercase letters indicate significant difference between different P conditions and different genotypes (P < 0.05), error bars represent standard error of the mean (n = 3), according to Duncan’s multiple-range test, the below is same. The P values of ANOVAs of P level (P), Genotype (G), and their interactions (P×G) were also shown (*P < 0.05, ns: not significant), the below is same.


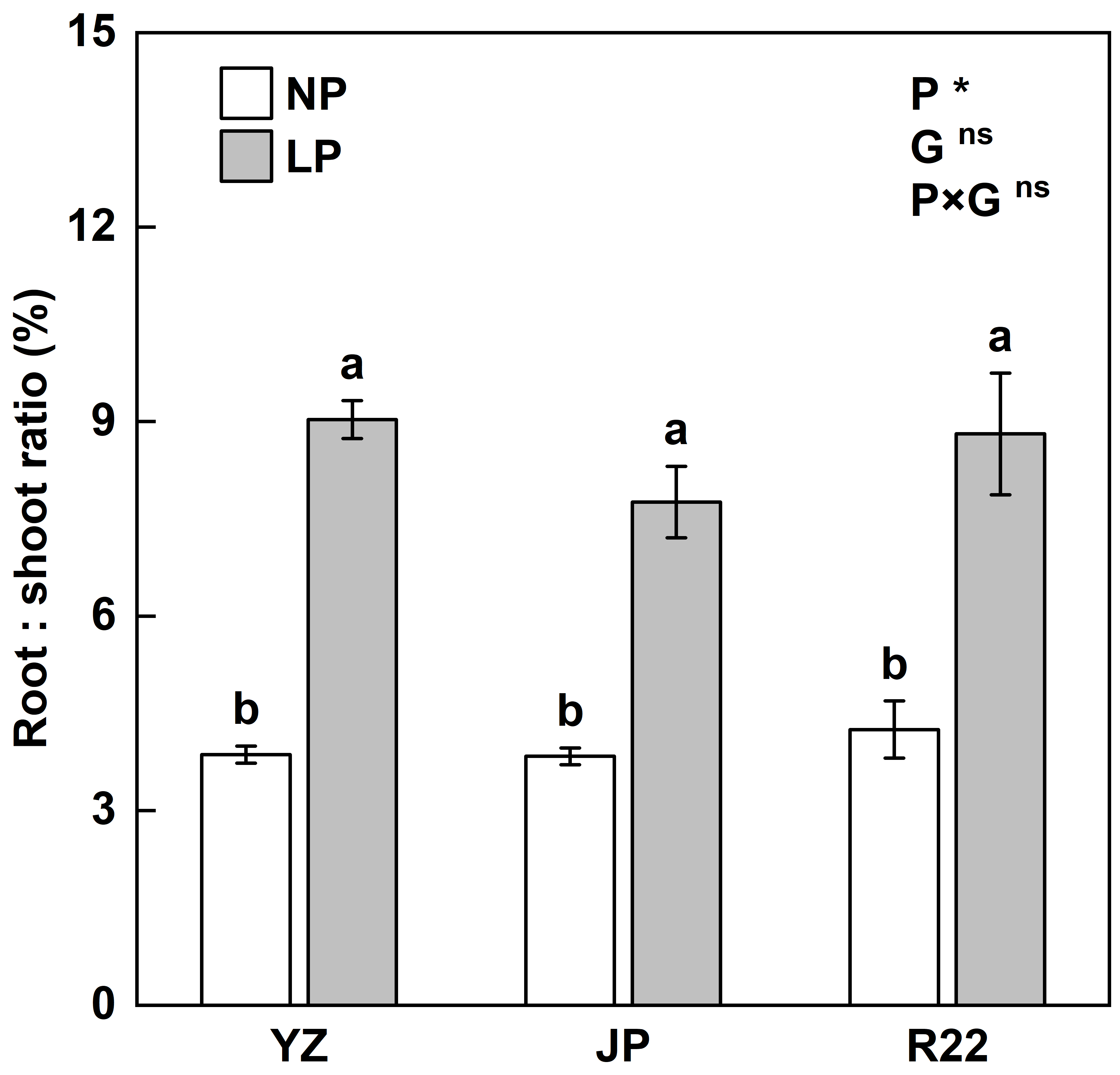


**Supplementary Figure 6.** The root: shoot ratio of the three sugarcane genotypes under the LP and NP conditions.
